# Does googling for preconception care result in information consistent with international guidelines: a comparison of information found by Italian women of childbearing age and health professionals

**DOI:** 10.1186/1472-6947-13-14

**Published:** 2013-01-25

**Authors:** Eleonora Agricola, Francesco Gesualdo, Elisabetta Pandolfi, Michaela V Gonfiantini, Emanuela Carloni, Pierpaolo Mastroiacovo, Alberto E Tozzi

**Affiliations:** 1 Bambino Gesù Children’s Hospital IRCCS, Epidemiology, Unit Piazza S. Onofrio 4, 00165, Rome, Italy; 2 Alessandra Lisi International Centre on Birth Defects and Prematurity, Via Carlo Mirabello 14, 00192, Rome, Italy

**Keywords:** Preconception, Adverse pregnancy outcomes, Folic acid, Google, Internet, Health information

## Abstract

**Background:**

Preconception counseling is effective in reducing the risk of adverse pregnancy outcomes. The Internet is commonly used by women and health professionals to search for health information. We compared the consistency of preconception information found on the Internet with the recommendations published by American Journal of Obstetrics and Gynecology (AJOG) simulating a web search by women of childbearing age and health professionals.

**Methods:**

We reviewed websites resulting from a Google search performed using search strings selected by Italian women of childbearing age and health professionals. We investigated if retrieved information was consistent with AJOG recommendations for preconception care. Logistic regression was used to compare presence of consistent recommendations between women and health professionals.

**Results:**

The highest frequency of correct recommendations was found for *folic acid supplementation* (39.4% of websites). Consistency of preconception information did not significantly differ between search strategies except for *folic acid supplementation. “Communities and blogs”* website category provided less frequently correct recommendations compared with “*Medical/Public Agency"* category (i.e. *folic acid supplementation* (aOR 0.254; CI 0.098-0.664; p = <0.01). Commercial links, found in 60% of websites, were associated with presence of correct recommendations excepting few items (i.e. *physical exercise* (aOR 1.127; CI 0.331-3.840; p = 0.848).

**Conclusions:**

Preconception information found is poor and inaccurate regardless of the search is performed by women or health professionals. It is unlikely that information found on the web have any positive impact among women and health professionals in our setting. Strategies to improve preconception information on the web and education of health professionals for web searching of health information should be considered.

## Background

Maternal health, appropriate lifestyle such as smoking and drinking cessation, folic acid supplementation and adoption of an appropriate diet before and at the time of conception are positively associated with fetal health and pregnancy outcomes [1-8]. Many conditions can be prevented through appropriate preconception counseling including i.e. infertility, miscarriage, gestational diabetes, pre-eclampsia, placenta abruption, birth defects and malformations, fetal restriction growth, low birth weight and prematurity.

International Health Agencies have therefore issued recommendations on preconception care indicating that prevention before conception is a crucial tool for identifying, modifying and reducing the risk factors for adverse pregnancy outcomes in women of childbearing age [1,6,9,10].

Although recommended, preconceptional counseling is still infrequently practiced by women of childbearing age and rarely offered by healthcare professionals. On the other hand a large proportion of women search for information regarding fertility and pregnancy on the web [11]. Moreover, pregnant women use the Internet in order to gather information, make decisions, and get support on pregnancy by participating in social networks [12-14]. Along with patients, also physicians consult the Web to search for health information. They frequently use generic search engines, like Google, to improve their knowledge, quickly respond to patients’ questions and make rapid diagnoses [15-19]. Recent surveys show that information found on the Internet influence physicians’ behavior and their relationship with patients [20-22].

Quality of information found on the web may largely vary and Internet users may be misled by false or incomplete information regarding health [23].

Official surveys report that in 2010 60.1% of the population over the age of 15 use the Internet in the USA [24], while in 2011 56% of European population used it daily [25].

According to a report issued in 2010 by the Italian Institute of Statistics (ISTAT), in Italy Internet use is 48.9% in the population over the age of 6. Up to 87% of women of childbearing age navigate the Internet, and 46.5% of them use the web to search for health information [26]. An European survey on generic medical practices conducted in 2007 showed that 70.7% of Italian general practitioners use the Internet and 81.4% have a computer in their consultation room [27]. Moreover, a study performed in 2011 on Italian pediatricians showed that 71.9% of them went online during their practice [20].

It is likely that patients and health professionals use different approaches in searching information on preconception care through the Internet and it is not known if different search strategies result in different level of consistency with current recommendations.

We therefore conducted a study to compare the frequency of web information on preconception care consistent with current guidelines as if a web search on this topic was performed by women of childbearing age who plan a pregnancy or by health professionals.

## Methods

In this study we reviewed a group of websites found through a number of simulated web searches on Google by Italian users.

### Search strings selection

By means of separate interviews, we investigated the likely search strings for a web search on preconception care in two groups of individuals: 21 women planning a pregnancy and 18 health care professionals, including gynecologists, pediatricians, neonatologists and geneticists. Mean age of the interviewed women was 32.02 years, 80% of them reported to be employed while 65% had a University degree. The interviews were conducted by email. Interviewees were invited to provide at least two search strings that they have used to perform a web search on preconception topics. Respectively, the two groups generated a list of 35 and 38 search strings excluding double entries.

We then submitted each of the strings into Google Adwords (Keyword Tool), which is a tool that reports the actual web search volume for a specific country in a specific period of time (Table 1) [28]. Based on the obtained search volume ranks, we selected the 12 strings with the highest volume score for each group (women and health professionals: 24 total search strings).

**Table 1:** List of search strings chosen by women of childbearing age and health professionals and the corresponding Google Adwords (Keywords Tool) search volumes

**Search strings of women of childbearing age**	**Google Adwords Keyword Tool search volumes**
Pregnancy	3350000
Mums	550000
Menstruation	550000
Ovulation	301000
Maternity	246000
To get pregnant	165000
Conception	135000
Fertile period	90500
Amniocentesis	49500
To have a baby	22200
Genetic diseases	8100
Check up before pregnancy	720
**Search strings of health professionals**	**Google Adwords Keyword Tool search volumes**
Toxoplasmosis	49500
Genetic diseases	8100
Preconception	1900
Folic acid before conception	1000
Check up before conception	480
Reproductive health	110
Pre-conception drugs	46
Pre-conception lifestyle	-*
Pre-conception obesity	-*
Pre-conception prevention	-*
Pre-conception chickenpox	-*
Pre-conception rubella	-*

*Key of Google Adwords (Keyword Tool) explaining dash: “The approximate 12-month average of the number of searches on Google for each keyword in the locations, languages, and devices that you selected. If you’ve selected multiple locations, a location that isn’t a country, or if we don’t have enough data, you’ll see this noted with a dash (−)” (28).

### Search and selection of websites

We launched 24 searches on Google using each of the selected search strings in a single session on October 4th, 2011. For each search, the first search engine result page (SERP) for each search string was saved on a hard disk for off line review (list available on request). Each SERP showed a total of 10 websites. On the same day, the web pages were saved on a hard disk using an offline browser and limiting the sublink download to a depth of three levels. The sponsored websites appearing on the SERP were not included in the analysis.

### Review process

In order to analyze the information quality on preconception care, we created a checklist of 19 items summarizing recommendations published by the American Journal of Obstetrics and Gynecology (AJOG) [2,9].

The review process for each item was conducted to find out whether it corresponded to: a) absent, incorrect or incomplete recommendation; or b) correct and complete recommendation. The web pages resulting from the searches were reviewed by a single operator (EA) based on the checklist.

We recorded the type of website choosing among five categories, as follows: a) Medical/Public Agencies: web pages with a clear affiliation to a hospital, a University, a medical centre, or a national health agency; b) Communities and blogs: websites managed and maintained by independent groups, where an interactive discussion with external users is displayed (social networks were not included in this category) c) Magazines: news magazine websites; d) Wikipedia: web pages belonging to the web encyclopedia; and e) Others: web pages not classifiable in the previous categories, including YouTube and sites belonging to any brand or industry.

### Statistical analysis

We estimated that 182 websites (91 per group) were needed for finding significant differences in consistent recommendations with guidelines between the simulated search performed by women and the one performed by health care professionals, with the following assumptions: 80% power, 5% significance level and an expected frequency of correct recommendations of 20% in searches performed by women and of 40% in searches performed by health care professionals. We took into account a potential number of double entries of 30%, due to website overlapping across searches conducted with different keywords, yielding a total sample size of 236.

We described the frequency of recommendations by group (women or health professionals) and we used the chi square test for comparing proportions. We performed logistic regression models to assess the effect of search strategy on presence of a recommendation consistent with AJOG guidelines [2], adjusted for website category and presence of commercial links. We considered results significant when p-values were below or equal to 0.05 for both the univariate and multivariate analyses.

We used STATA 11 statistical software for analysis.

## Results

From a total of 240 web pages (2 groups, 12 search strings per group, 10 web pages for each first SERP), 14 websites (5.8%) were excluded because they were double entries within each search group. The remaining 226 were included in the analysis. Only 15 websites (6.6%) overlapped between the search by women and the one by health professionals.

Table 2 shows the distribution of web categories by search group. The “*Communities and blogs*” category was the most represented overall; no social network website resulted from the simulated search. The second most frequent category was *“Medical/Public Agency”* while “*Magazines*” and “*Wikipedia”* were less frequent. Websites in the “*Medical/Public Agency”* category were three times more frequent in the health professional searches than in the women searches, while the “*Communities and blogs”* category was more frequently represented in the women searches.

**Table 2:** Distribution of websites by category and search groups

	**Search group**			
	**Health care professionals**	**Women planning a pregnancy**		**Total**
**Website category**	**N (%)**	**N (%)**	**p value**	**N (%)**
Public health agency, university, research entity	40 (36.4)	12 (10.3)	<0.01	52 (23.0)
Communities and blogs	47 (42.7)	72 (62.1)	<0.01	119 (52.7)
Magazines	6 (5.5)	7 (6.0)	NS	13 (5.8)
Wikipedia	3 (2.7)	9 (7.8)	NS	12 (5.3)
Others	14 (12.7)	16 (13.8)	NS	30 (13.3)
**Total**	**110 (100)**	**116 (100)**		**226 (100)**

Regarding the frequency of correct preconception recommendations, we found that they were infrequently reported regardless of the search strategy (Table 3). The most frequent recommendations consistent with AJOG guidelines were those regarding *folic acid supplementation, rubella immunization*, *smoking cessation*, *doctor’s appointment for preconception counseling,* and *doctor’s advice for drug assumption*. The least represented information were those about *asthma control*, *avoiding contact with toxics and pesticides*, and *pertussis immunization*. According to the multivariate analysis, correct recommendation on folic acid supplementation was found more frequently in searches performed by health professionals compared with women (Table 3). Moreover, most of the correct recommendations were significantly less represented in websites belonging to the *Communities and blog* category compared with those belonging to the *Medical/Public Agency* category: *folic acid supplementation* (aOR 0.254; CI 0.098-0.664; p = <0.01)*, medical conditions control* (aOR 0.141; CI 0.040-0.493; p < 0.01)*, vaccination status review* (aOR 0.115; CI 0.024- 0.564; p < 0.01)*, doctor’s appointment for preconception counseling* (aOR 0.141; CI 0.037-0.546; p < 0.01).

**Table 3:** Distribution of correct recommendations by search group, univariate and multivariate analysis

	**Search group**			**Univariate analysis**			**Multivariate analysis**		
** Recommendation**	**Health care professionals N (%)**	**Women planning a pregnancy N (%)**	**Total (%)**	**OR**	**95% CI**	**p**	**aOR**	**95% CI**	**p**
Take folic acid	55 (50.0)	34 (29.3)	89 (39.4)	2.41	1.35-4.33	0.001	2.064	1.129 – 3.771	0.018
Control medical conditions	29 (26.4)	31 (26.7)	60 (26.5)	0.98	0.52-1.85	0.951	0.929	0.470 - 1.836	0.832
Control asthma	17 (15.7)	7 (6.1)	24 (10.8)	2.88	1.07-8.06	0.020	2.329	0.867 – 6.255	0.094
Control diabetes	30 (27.3)	27 (23.3)	57 (25.2)	1.24	0.65-2.36	0.489	1.065	0.546 - 2.075	0.854
Control epilepsy	22 (20.2)	10 (8.6)	32 (14.2)	2.68	1.13-6.45	0.013	2.117	0.874 - 5.129	0.097
Go on diet if overweight or obese	34 (31.2)	24 (20.7)	58 (25.8)	1.74	0.91-3.33	0.072	1.823	0.924 - 3.599	0.083
Review vaccination status	20 (18.2)	27 (23.3)	47 (20.8)	0.73	0.36-1.47	0.346	0.739	0.342 - 1.595	0.441
Verify immunity against rubella and receive immunization if susceptible	45 (40.9)	38 (32.8)	83 (36.7)	1.42	0.80-2.54	0.204	1.347	0.702 - 2.584	0.370
Verify immunity against chicken pox and receive immunization if susceptible	26 (23.6)	25 (21.6)	51 (22.6)	1.13	0.58-2.20	0.708	0.793	0.386 - 1.629	0.528
Verify immunity against hepatitis B and receive immunization if susceptible and not previously vaccinated	15 (13.6)	13 (11.2)	28 (12.4)	1.25	0.53-2.97	0.580	1.185	0.504 – 2.786	0.698
Verify pertussis immunization record and receive immunization if last dose >10 years before	5 (4.5)	3 (2.6)	8 (3.5)	1.79	0.36-9.74	0.490	1.244	0.254 – 6.103	0.788
Ask advice to doctor about administration of any drug	35 (31.8)	34 (29.3)	69 (30.5)	1.13	0.61-2.06	0.682	1.255	0.657 - 2.397	0.491
Make an appointment with the doctor for preconceptional counseling	32 (29.1)	41 (35.3)	73 (32.3)	0.75	0.41-1.36	0.315	0.732	0.377 - 1.424	0.359
Stop smoking	36 (32.7)	38 (32.8)	74 (32.7)	1.00	0.55-1.81	0.996	0.990	0.515 – 1.901	0.976
Stop drinking alcohol	29 (26.4)	30 (25.9)	59 (26.1)	1.03	0.54-1.94	0.932	0.994	0.512 – 1.930	0.985
Stop illicit drug use	19 (17.3)	10 (8.6)	29 (12.8)	2.21	0.92-5.41	0.052	1.655	0.684 - 4.003	0.263
Do physical exercise	14 (12.7)	11 (9.5)	25 (11.1)	1.39	0.56-3.48	0.437	1.013	0.399 - 2.573	0.978
Avoid contacts with toxics and pesticides	6 (5.5)	7 (6.0)	13 (5.8)	0.90	0.26-3.10	0.852	0.689	0.200 - 2.377	0.555
Verify immunity against toxoplasmosis and adopt preventive behaviors if susceptible	38 (34.5)	26 (22.4)	64 (28.3)	1.83	0.98-3.43	0.043	1.808	0.947 - 3.452	0.073

We found that 60.0% of the websites presented commercial links; in particular, women’s search strategy selected 68.3% websites containing a commercial link, compared with 51.7% obtained through health professionals’ search strings (p = 0.008). Three websites were explicitly branded by pharmaceutical industries.

According to the multivariate analysis, only the least represented items were not positively associated with the presence of a commercial links [*asthma control* (aOR 2.734; CI 0.854-8.756; p = 0.09)*, pertussis immunization* (aOR 0.989; CI 0.131-7.467; p = 0.992)*, illicit drug cessation* (aOR 2.350; CI 0.787-7.020; p = 0.126)*, physical exercise* (aOR 1.127; CI 0.331-3.840; p = 0.848)*, avoiding toxics and pesticides* (aOR 1.212; CI 0.235-6.261; p = 0.818)].

## Discussion

The likelihood of finding preconception care recommendations consistent with AJOG on the Internet was low, using search strings selected either by health professionals or by women of childbearing age.

This study shows that current approaches for searching preconception information on the web do not result in finding a complete and accurate set of recommendations, although we found no websites reporting totally incorrect preconception information. The obtained result is helpful to estimate the level of consistency of information found on the web considering the increasing use of the Internet for seeking health information by women and health care professional, and the importance of the web as a helpful instrument for health professionals for rapidly improving their knowledge and communicating with patients [11-22].

The recommendation on *folic acid supplementation* was, overall, the most frequently found on the Internet; moreover, it was significantly easier to find a correct recommendation on this subject for a health professional than for a woman of childbearing age. This result may be explained by the observation that a specific string search on this topic was provided by health professionals and not by women. However other specific search strings, as those on lifestyle and on preconceptional visit, were evenly distributed between the search groups and did not result in any difference on correct recommendation. On the other hand the high frequency of correct recommendations on folic acid supplementation may be favored by a recent campaign conducted in Italy on folic acid supplementation [29-31].

Since physicians may be influenced in their clinical decisions by information found through Google searches, they should be warned on the frequent inconsistency of data retrieved by this means. Information obtained from general search engines should always be integrated with recommendations obtained from specific medical databases. This observation seems particularly relevant, as Google is planning to release utilities such as Google symptoms [32], that may be used by physicians in the near future.

Information on *diet if overweight or obesity* were correctly reported only in 1/4 of the reviewed websites. The insufficient presence of this recommendation, in particular on websites found by women of childbearing age, parallels the results from other studies that show a poor quality of nutritional information on the Internet for patients with specific medical conditions [33-35].

As for recommendations regarding vaccinations, *rubella immunization* is the second most frequently found item consistent with international recommendations, with no significant difference by search group (health professionals vs women of childbearing age). This result can be explained by the fact that rubella is a widely known risk factor for fetal disease. Rubella infection still presents a worrying incidence [36], and vaccination against rubella is included in an international eradication program [37]. Moreover, Italy has adopted a national plan for preconception health care promoting medical check-ups including blood tests to verify immunity against rubella [38]. Therefore, health professionals and women in childbearing age are widely informed on rubella’s risks for pregnancy and on measures for its prevention.

On the contrary, the recommendation regarding *pertussis immunization* is one of the less represented. This finding reflects the fact that pertussis is not sufficiently recognized as a threat for newborns. In several outbreaks the maternal-newborn transmission has been observed as one of the main transmission patterns [39].

Regarding web page categories, *Communities and blogs* were more frequently retrieved by women, while *Medical/Public Agencies* were the most frequently found by health professionals. This is strongly related to the source of the search strings used for the search (respectively, women and health professional). *Communities and blogs* represents overall the most represented category. This is an interesting result, as it shows a general interest among Internet users for the preconception care topic. Although, it is also the website category that reports less information consistent with international guidelines. This result suggests that preconception care specialists should consider an active use of blogs, forums and other social networks as powerful media for providing women of childbearing age with correct preconception information.

Presence of commercial links in a web page was associated with a higher quality of preconception information. This result shows that industries are actually implementing promotion of health care on the web as a part of their marketing strategy. Among the websites that were reviewed, few were disclosed as property of pharmaceutical industries. We cannot exclude that other websites were driven by other companies.

### Strengths and limitations of the study

The main strength of our study concerns the methodology: we investigated preconception information available on the Internet through a formal approach that helps avoiding the biases originating from arbitrary choices of keywords by study researchers. Several authors investigated quality of information found on the Internet regarding specific health topics, including cystic fibrosis [40], pediatric cancer [41], rheumatologic diseases [42], Mediterranean diet [43]. The search keywords used in these studies were chosen by the authors themselves, either simulating a search by a random user, or simulating a patient’s experience [44]. Weighing suggestions from a group of users against the frequency of Google searches should increase the likelihood to perform simulations closer to real life. We recommend this methodology for the evaluation of quality of medical information on the Internet.

The main limitation of our study is that it has a cross sectional design, and it analyses a static situation at the moment when the survey has been conducted. Presence of information on the Internet, on the contrary, is intrinsically dynamic. A periodic survey of the web would constitute a precious, alternative source of information on specific public health issues.

The analysis has been conducted using only Italian search strings, therefore the results may not be generalizable to other countries. Finally, we investigated presence of specific web information using generic search strings. This may have led to an underestimation of the presence of consistent recommendations on the web. Nevertheless, we can assume that this approach reflects the average search strategy most frequently chosen by the users.

## Conclusion

The present study results indicate that a strong effort is needed for implementing web communication strategies regarding preconception health, in order to guarantee a correct dissemination of recommendations consistent with guidelines.

To this aim, a successful strategy would imply investigating the most frequently used search strings, and subsequently improving the rank of websites providing correct and complete preconception recommendations through a thorough Search Engine Optimization (SEO). An additional effort should be put in the dissemination of consistent information to websites that are not directly managed by public health agencies. From this point of view, engagement in social networking and communities, being the latter the category of websites most frequently found by women, should be implemented.

With regard to health professionals, this study shows that a deep and constant update on search strategies for health information should be included in medical education, in order to avoid incurring in unreliable or incomplete information.

With regard to women of childbearing age, the general inconsistency of web information on preconception care confirms the crucial importance of having a physician appointment before conception in order to receive a correct preconception counseling.

In conclusion, dissemination of information on the web can be a powerful tool to guide proper preventative actions in the preconception period. Although we did explore the Italian setting only, it is likely that specific web strategies for disseminating appropriate preconception recommendations are still largely insufficient also in other settings. Studies like the one described here may help improving penetration of information and ultimately supporting healthy lifestyles aimed to prevent adverse pregnancy outcomes.

## Competing interests

The authors declare that they have no competing interests.

## Authors’ contributions

EA coordinated the study, designed the study and participated in the writing process and in the data review. FG designed the study and drafted the manuscript. EP conducted the interviews. MVG conducted the interviews and revised the final version of the manuscript. EC performed the statistical analysis. PM conceived the study, participated in its design, AET conceived the study, participated in its design and coordination and drafted the manuscript. All authors read and approved the final manuscript.

## Pre-publication history

The pre-publication history for this paper can be accessed here:

http://www.biomedcentral.com/1472-6947/13/14/prepub
